# Bilateral ‘kissing’ molars: A case report

**DOI:** 10.1002/ccr3.6407

**Published:** 2022-11-12

**Authors:** Bilal Aslam‐Pervez, Emma Carr

**Affiliations:** ^1^ Oral and Maxillofacial Surgery, NHS Greater Glasgow and Clyde Queen Elizabeth University Hospital Glasgow UK

**Keywords:** anaethesia, extraction, local anesthetic, molar, paraesthesia, risk, surgery, tooth follicle

## Abstract

‘Kissing molars’, were described by Van Hoof in 1973 as when the occlusal surfaces of impacted molars are united by the same follicular space and the roots point in the opposite direction. There are very few published cases in the literature, and it is an extremely rare form of impaction.

## Abstract 2

This manuscript concerns the diagnosis and management of a rare dentoalveolar clinical and radiographic presentation, which may be encountered by and be of interest to dental practitioners in both primary and secondary care.

## 1 INTRODUCTION

Kissing molars are a rare example of impaction wherein the “occlusal surfaces of impacted molars are united by the same follicular space and the roots point in the opposite direction”.^1^ Most commonly, they involve mandibular third (M3Ms) and second molars; however, other teeth have been known to be involved, for instance a third and fourth supernumerary mandibular molar.^2^

Impaction affects as many as 72.7% of third permanent molars in 20–30 years olds.^3^ There are many known causes of failure of eruption of teeth such as trauma to primary dentition, mechanical failure of eruption, isolated ankylosis, and lack of space.^4^ It is already known that certain medical conditions can predispose to failure of eruption of teeth such as cleidocranial dysostosis.^5^

Very few bilateral cases have been published in the literature, so this case adds to this small cohort with the hope to increase awareness and ultimately optimize confidence for onward referral and management. We will also discuss possible treatment planning options for its management.

## 2 CASE REPORT

A 20‐year‐old male presented to Glasgow Dental Hospital in August 2011 following a referral from a general dental practice in London. He presented with a dull ache, present for the past 12 months, arising from the left angle of the mandible. The patient brought with him an orthopantomograph (OPG) taken in Christmas 2010. Medically, the patient was fit and well, with no known drug allergies and taking no regular medications. He had a 1‐pack year smoking history (10 cigarettes on a daily basis for the past 2 years) and consumed approximately 14 units of alcohol per week.

Clinical examination did not reveal any facial asymmetry or significant extra‐oral findings. Intra‐orally, the soft tissues were normal. There were seven unerupted teeth in total (UR7, UR8, UL8, LL7, LL8, LR7, and LR8). The upper left second premolar was found to be missing.

**FIGURE 1:**
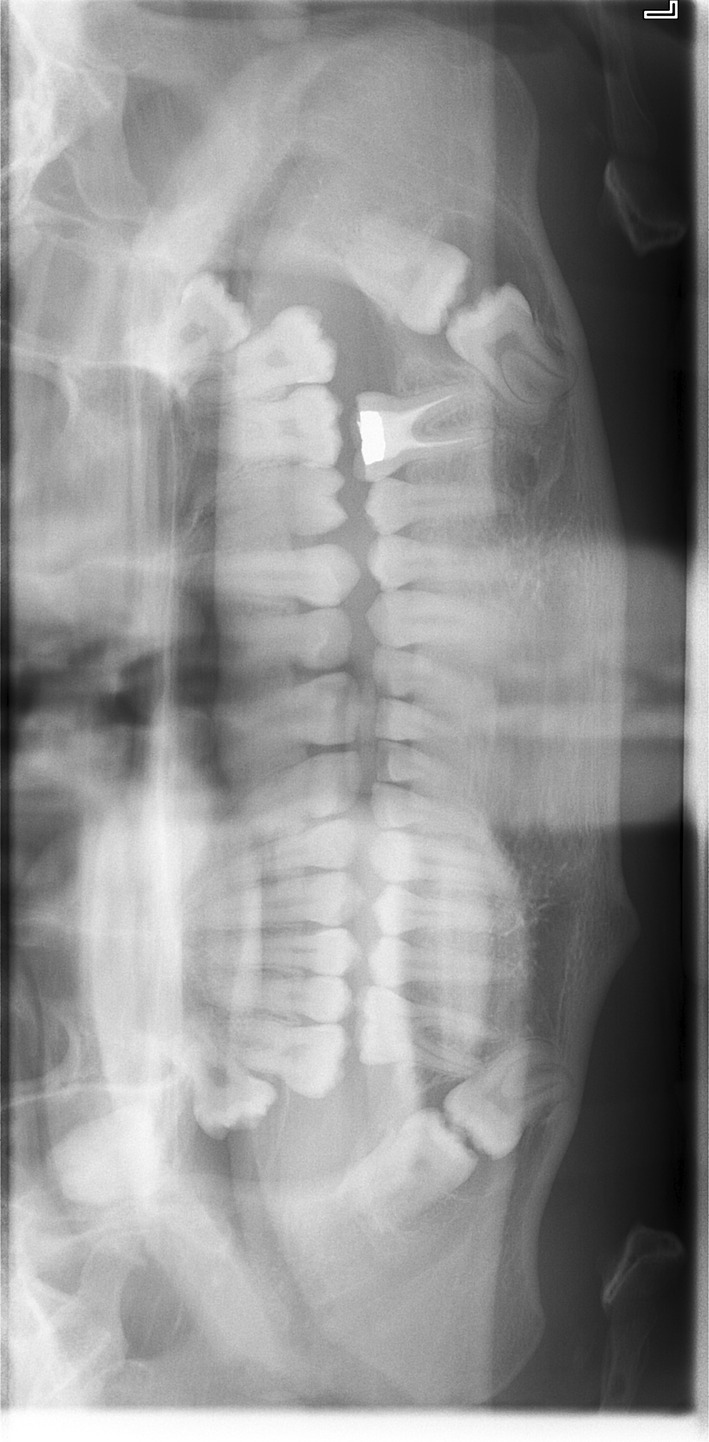
Orthopantomograph

The orthopantomograph (Figure 1 *)* shows presence of bilateral ‘kissing molars’ in the lower quadrants associated with cystic lesions encompassing the molar crowns. An ectopic UR8 was also noted with an associated mass in the right maxillary antrum.

The patient was advised to have a CBCT taken to determine the risks of surgical removal of these teeth. A report mentioned the following relevant features:

1. There is a 17 mm vertical × 18 mm medio lateral × 24 mm AP corticated soft tissue mass in the right maxillary sinus. This is intimately involved with a displaced UR8.
2. There is thinning of the lateral wall of the right maxillary sinus. The vertical dimension is actually in excess of 28 mm, and this mass involves the crowns of both UR8 and UR7.
3. UR8 – the apices of this tooth are dilacerated and engage the lateral wall of the right maxillary sinus. They abut the pterygoid palatine fossa.
4. The roots of UR7 engage both the floor of the maxillary sinus and the floor of the right nasal cavity (palatal root),
5. The roots of UR7 lie between the roots of UR6. There is apical root resorption of the distobuccal root of UR6 by this arrangement ‐ blunting of the palatal root of UR6 is also noted.
6. UR5 is two‐rooted.
7. UL8 has dilacerated roots and engages the floor of the left maxillary sinus.
8. UL4 is rotated and 25 is missing.
9. Both LR8 and LR7 show a ‘kissing molar’ arrangement, with follicular expansion measuring up to 5 mm.
10. LR8 – the roots engage the lingual cortex and are dilacerated.
11. LR7 – the roots engage the inferior cortex. The roots of LR7 displace the right ID canal lingually.
12. Both LL7 and LL8 also show a ‘kissing molar’ arrangement, again with follicular expansion in a similar pattern to the contra‐lateral side
13. The right ID canal takes a lingual course through the ramus of the mandible, and it then runs lateral to and inferior to the expanded follicle before being displaced lingually by the crown and root structure of LR7. It is intimately involved with the apices of LR7, rounding its apex to emerge as the right mental foramen.
14. The left ID canal takes a lingual course through the ramus of the mandible before being displaced inferior by the follicle of the kissing molar arrangement. It then swings lingually to be in direct contact with the roots of LL7 before rounding the apices to emerge as the left mental foramen.
15. The apices of both LL7 and LR7 project as far forward as the ‘mental foramena’.

**FIGURE 2:**
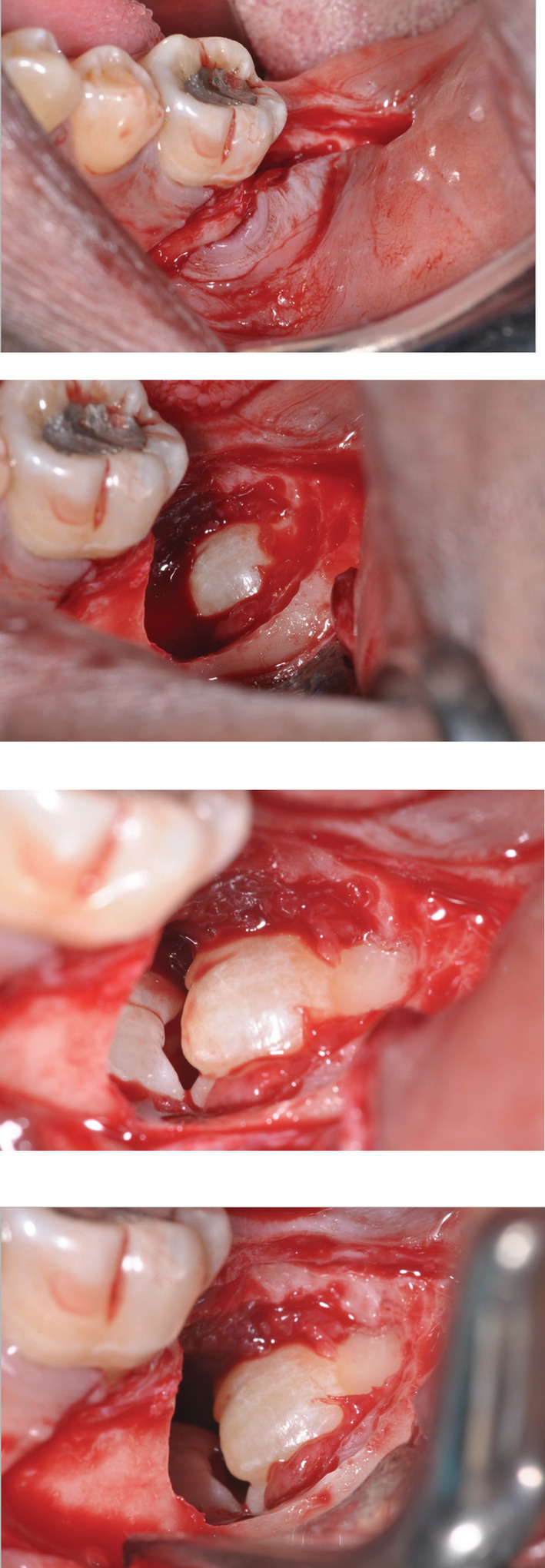
Clinical photographs taken during surgical removal of kissing molars

The patient underwent a biopsy and marsupialization under local anesthetic of the cystic lesion in the lower left quadrant. This was confirmed as a dentigerous cyst, and histopathology report can be found in Box 1. Surgical clinical photographs can be seen Figure 2.

BOX 1 CLINICAL HISTORY:

? Dentigerous cyst? odontogenic keratocyst. Specimen 1 – tooth lower left 8 with cyst lining. Specimen 2 – soft tissue at bone of cyst cavity (? cyst or nerve). Specimen 3 – soft tissue overlying retromolar pad (? cyst or mucosa). Specimen 4 – upper right 8 tooth with? dentigerous cyst.

MACRO:

A: dental cyst ‐ left tooth 8.

A 15 × 10 × 8mm tooth with 20 × 10 × 3mm surrounding soft tissue.

B: Dental biopsy ‐? cyst or nerve left cavity.

A 15 × 2 × 2mm piece of fibrous tissue.

C: Dental biopsy – left retro molar pad.

Two pieces of tissue, 12 × 5 × 5mm and 10 × 5 × 2mm.

D: Dental cyst – upper right 8.

A 20 × 12 × 7mm tooth with ruptured cyst, 30 mm in diameter.

MICROSCOPY:

A: Microscopy shows chronically inflamed and scarred fibrous connective tissue which is partially covered by a spongiotic and inflamed non‐keratinising stratified squamous epithelium.

The appearances are consistent with the lining from an inflamed dentigerous cyst. There are no features to suggest an odontogenic keratocyst.

B: Microscopy shows fibrous connective tissue containing bone fragments. There is focal mild non‐specific chronic inflammation and a small amount of haemosiderin. No epithelium is included.

The appearances are those of fibrous connective tissue with bone and patch. non‐specific chronic inflammation. There are possibly small nerves within the Tissue; however, there are no large nerves.

C: Microscopy shows fibrous connective tissue covered by the stratified squamous epithelium. There is focal surface parakeratosis. There is also patchy mild spongiosis and inflammation of the squamous epithelium. The underlying tissue contains a patchy non‐specific chronic inflammatory cell infiltrate. There is also a small amount of bone associated with bacterial colonies.

The appearances are consistent with focally inflamed retromolar squamous mucosa. There are no definite features to suggest a cyst.

D: Microscopy shows a fibrous walled cyst lined by a non‐keratinising stratified squamous epithelium. There is bone within the outer aspect of the cyst wall.

The appearances are consistent with a simple dentigerous cyst. There are no features to suggest an odontogenic keratocyst.

There is no evidence of atypia or malignancy in any of the biopsies.

The definitive treatment options considered were as follows:

- Surgical removal of the lower 7 s and 8 s +/− UR8 under general anesthetic
- Surgical removal of lower left second and third molars
- Coronectomies of involved molars (first suggested at initial visit, but an unlikely long‐term solution)
- Monitoring of conditions with no active surgical intervention.

After discussion with the patient, it was clear that he was keen on having surgical intervention due to the severity of the symptoms. A referral was made to the local Oral and Maxillofacial unit with a provisional plan to surgically extract the three pathologies and associated teeth.

On further discussion with the Oral and Maxillofacial consultant, the treatment plan was modified to extract only the upper right 7 +/− 8 if visible and the lower left kissing molars only (which had an open oral communication following marsupialisaton) due to the risk of damage to the inferior alveolar nerves.

Surgical extractions of the teeth were arranged under a general anesthetic. The lower left kissing molars were extracted using a three‐sided mucoperiosteal flap. This involved careful bone removal followed by methodical sectioning of the crown and roots and finished with curettage and enucleation of the cyst, making sure the inferior alveolar nerve and bone were preserved to avoid paraesthesia and mandibular fracture, respectively. Throughout the procedure, the inferior alveolar nerve was not exposed.

The upper right molars were removed via a two‐sided mucoperiosteal flap and bone removed with caution to keep the maxillary antrum lining intact. The cyst lining was punctured, releasing a yellow fluid. The splayed roots of the upper right 7 proved difficult; however, they were extracted successfully following sectioning and elevation. The resulting oro‐antral communication from removal of the teeth was closed with tissue from the buccal fat pad, and a 2‐layered closure technique was employed. The patient was commenced on an antral regime following recovery to avoid an oroantral fistula formation.

An uneventful recovery followed postoperatively, with simple analgesics and chlorhexidine use. The patient reported no paraesthesia and did not report any symptoms of dry socket occurring. At a 3‐month review, complete soft tissue healing had occurred and the patient reported no problems. Histology reports confirmed the diagnoses of a dentigerous cyst (*see* Box 1). A 1‐year review was arranged to ensure bony infill of the areas and monitor the lower right quadrant which was left untouched.

## 3 DISCUSSION

There remains no concrete evidence regarding the etiology of kissing molars. It is theorized by some that an ectopic tooth bud is responsible and that early cystic development around a molar can cause their crowns to displace^6^ mucopolysaccharidosis^7^ and hyperplastic dental follicles^8^ has also been suggested.

Impaction of teeth is a common presentation that dentists and surgeons routinely diagnose. Most commonly, this involves mandibular third molars impacting against the adjacent second molar. When partially erupted, this can lead to recurrent infections involving the pericoronal space. Cyst development can also be a complication of impaction, sometimes causing displacement of the tooth and adjacent structures. Our case was particularly interesting, not only due to its kissing molar formation, but because it was bilateral in nature. Currently, only nine other cases of bilateral kissing molars could be found in the literature‐ see Table 1.

**TABLE 1:** Summary of cases of bilateral kissing molars within literature

Study authors	Date of publication	Journal published	Age and sex of the patient at presentation	Initial symptoms	Treatment	Associated pathology
Zerener et al.^9^	2016	*Case Reports in Dentistry*	38F	Swelling at right lower angle of the mandible	Surgical Removal of kissing molars	Nil
Sarna er al.^10^	2021	*Clinical Case Reports*	27 M	Severe pain at left angle of the mandible	Conservative	Nil
Anish et al.^11^	2015	*Clinical Practice*	35 M	Disloged filling on the upper right molar	Conservative	Nil
Bakaeen et al.^12^	2004	*British Journal of Oral and Maxillofacial Surgery*	23 M	Facial pain	Surgical Removal under GA	Nil
Kiran et al.^8^	2014	*Chinese Journal of Dental Research*	18F	Bilateral diffuse swelling of the mandible	Surgical Removal under GA	Nil
Nakamura et al.^13^	1992	*Dento Maxillofacial Radiology*	25 M	Asymptomatic	Conservative	Mucopolysacchariidosis
Nakamura et al.^13^	1992	*Dento Maxillofacial Radiology*	17 M	Aymptomatic	Conservative	Mucopolysacchariidosis
Nakamura et al.^13^	1992	*Dento Maxillofacial Radiology*	21 M	Asymptomatic	Conservative	Mucopolysacchariidosis
Robinson et al.^14^	1991	*Oral Surgery Oral Medicine, Oral Pathology*	25 M	Asymptomatic	Conservative	Nil

This case highlighted how there is no set protocol or guidelines in place for the management of kissing molars. Some cases may present a high risk of pathological fracture and inferior alveolar nerve injury. Radiographic features which would indicate a high risk of Inferior alveolar nerve (IAN) damage are deflection of the roots, narrowing of the roots, diversion of the canal, and narrowing of the canal. The risk factors which were deemed significant were darkening of the root, root deflection, dark and bifid apices of the roots, and narrowing of the canal^.^ ^15^ In these cases, a cone beam CT can prove helpful to provide information regarding the buccolingual positioning of the IAN, width of the remaining bone, and therefore aid planning.^16^ For our patient, the risk of damage to bilateral inferior alveolar nerves was increased due to their close proximity to the second molars. Therefore, following discussion with the patient, it was decided that surgical intervention was only to be carried out in the lower left quadrant where the dentigerous cyst was more advanced and presented a higher risk of infection due to its oral communication.

For some patients, orthodontic alignment of the impacted molars may be possible so an orthodontic opinion should be sought where appropriate. Alternatively, kissing molars can be monitored, but a discussion with the patient regarding the risks of nearby root resorption and cyst formation should be balanced against the risks of surgical intervention.

Some clinicians routinely prescribe post‐operative steroids following surgical removal of kissing molars.^2^ Improved patient comfort and reduced swelling and trismus following third molar removal and post operative steroid use have also been reported.^17^

## 4 CONCLUSION

Kissing molars present a challenging problem and must be treated on a case‐by‐case basis. Surgical extraction is one option; however, great care must be taken due to the increased risk of inferior alveolar nerve paraesthesia. In some cases, regular monitoring of the teeth and associated follicles may be more appropriate.

Despite continuing research being needed to determine the etiology of this phenomenon, it is hoped that this case report will raise awareness and improve understanding of kissing molars so that clinicians can correctly diagnose, manage, or refer patients as appropriate.

## AUTHOR CONTRIBUTIONS

Bilal Aslam‐Pervez‐ Data collection, original draft manuscript, editing. Emma Carr‐ Draft manuscript, data collection and final editing.

## CONFLICT OF INTEREST

There are no conflicts of interest.

## ETHICAL APPROVAL

The authors state that this publication complies with ethical standards.

## CONSENT

Written consent was gained from the patient for the use of his clinical information, radiographic imaging, and photographs for publication.

## Data Availability

The data that support the findings of this study are available from the corresponding author upon reasonable request.
